# Amyotrophic Lateral Sclerosis (ALS): Stressed by Dysfunctional Mitochondria-Endoplasmic Reticulum Contacts (MERCs)

**DOI:** 10.3390/cells10071789

**Published:** 2021-07-15

**Authors:** Junsheng Chen, Arthur Bassot, Fabrizio Giuliani, Thomas Simmen

**Affiliations:** 1 Department of Cell Biology, Faculty of Medicine and Dentistry, University of Alberta, Edmonton, AB T6G2H7, Canada; junsheng@ualberta.ca (J.C.); bassot@ualberta.ca (A.B.); 2 Department of Medicine (Neurology), Faculty of Medicine and Dentistry, University of Alberta, Edmonton, AB T6G2H7, Canada; giuliani@ualberta.ca

**Keywords:** mitochondria-associated membranes (MAMs), amyotrophic lateral sclerosis (ALS), mitochondria-endoplasmic reticulum contacts (MERCs)

## Abstract

Amyotrophic lateral sclerosis (ALS) is a devastating neurodegenerative disease for which there is currently no cure. Progress in the characterization of other neurodegenerative mechanisms has shifted the spotlight onto an intracellular structure called mitochondria-endoplasmic reticulum (ER) contacts (MERCs) whose ER portion can be biochemically isolated as mitochondria-associated membranes (MAMs). Within the central nervous system (CNS), these structures control the metabolic output of mitochondria and keep sources of oxidative stress in check via autophagy. The most relevant MERC controllers in the ALS pathogenesis are vesicle-associated membrane protein-associated protein B (VAPB), a mitochondria-ER tether, and the ubiquitin-specific chaperone valosin containing protein (VCP). These two systems cooperate to maintain mitochondrial energy output and prevent oxidative stress. In ALS, mutant VAPB and VCP take a central position in the pathology through MERC dysfunction that ultimately alters or compromises mitochondrial bioenergetics. Intriguingly, both proteins are targets themselves of other ALS mutant proteins, including C9orf72, FUS, or TDP-43. Thus, a new picture emerges, where different triggers cause MERC dysfunction in ALS, subsequently leading to well-known pathological changes including endoplasmic reticulum (ER) stress, inflammation, and motor neuron death.

## 1. Introduction

Despite the ever-increasing knowledge about diseases affecting the central nervous system (CNS), neurodegenerative diseases have remained a stubborn health threat [1]. The major types of these diseases, including Alzheimer’s disease (AD), Parkinson’s disease (PD), and amyotrophic lateral sclerosis (ALS) share many symptoms at a cellular level, most notably the accumulation of unfolded protein aggregates [2]. Remarkably, some of the proteins that are subject to aggregation in these neurodegenerative diseases are observed in more than one condition, suggesting that neurodegenerative diseases share common mechanisms [3]. In some cases, such as Huntington’s disease (HD), these defects are easily traced to mutations in the genes encoding the proteins involved [4,5]. In other cases, most notably PD and ALS, aberrant protein folding can coincide with endoplasmic reticulum (ER) [6] and mitochondrial dysfunction [7,8]. This raises the possibility that localized protein aggregation in the cytosol could compromise the normal functions of other organelles. Often, this process is triggered with the oxidation of aggregation-prone protein monomers [9]. Subsequently, the entire cellular redox environment is altered and compromised across diverse organelles [10]. This is exemplified with the induction of the ER-associated unfolded protein response (UPR) and a more oxidizing ER lumen in the presence of cytoplasmic protein aggregates [11], which increases the chances for ER protein misfolding [12]. Subsequently, ER transmembrane sensor proteins like the inositol-requiring enzyme 1 (Ire1), an endonuclease and protein kinase R (PKR)-like ER kinase (PERK), ramp up the production of ER chaperones under the control of the X-box binding protein 1 (XBP1) transcription factor [13] or shut down ER protein synthesis, respectively [14].

However, in the case of ALS, the effects of cytoplasmic protein aggregates go beyond the ER and can also affect mitochondria, suggesting the overall relationship between organelles undergoes changes. This review will examine the nexus between cytosolic protein aggregation, mitochondrial dysfunction, and ER stress in motor neurons, where protein aggregates can compromise the communication on mitochondria-ER contacts (MERCs), triggering ALS. MERCs are amongst the best-characterized membrane contact sites (MCS), a type of intracellular structure that describes physical contacts between organellar membranes [15]. MCS are now known to form between essentially all cellular membranes [15,16]. Typically, these contacts do not form randomly, but rather assemble using tethering proteins and have key functions for the organelles involved. The best-characterized functions include the trafficking of lipids between organelles such as the exchange of phosphatidylinositol 4-phosphate and phosphatidylserine by oxysterol binding protein-related proteins 5 and 8 (ORP5, ORP8) at contact sites of the plasma membrane with the ER [17] or the transfer of Ca^2+^ ions between the ER and mitochondria via an interorganellar protein complex formed by ER inositol 1,4,5-trisphophate receptors (IP_3_Rs) and mitochondrial voltage-dependent anion channel 1 (VDAC1) [18]. Therefore, MCS determine organellar membrane properties and ion content. Moreover, emerging insight suggests that organellar membranes respond to reactive oxygen species (ROS) at the contact sites, as shown for instance by the ER transmembrane protein TMX1 that controls MERCs dependent on ROS and its thiol-containing thioredoxin domain [19].

At MERCs, ER and mitochondrial membranes approach each other at a proximity of 0–100 nm [20]. These contacts occur as sterol-enriched, raft-like rough ER (rER) wrapped around mitochondria (wrappER) at a 50 nm distance or as tighter, smooth mitochondria-associated membranes (MAMs), which sometimes manifest themselves as adhesion sites with direct contact between the two organelles [21]. Ongoing research aims to identify how these structures assemble with the help of protein tethers like the vesicle-associated membrane protein-associated protein B (VAPB) and protein tyrosine phosphatase interacting protein 51 (PTPIP51) [22,23] and with the help of regulatory proteins like phospho-furin acidic cluster sorting protein 2 (PACS-2) [24]. These tethering and regulatory proteins allow for extensive communication between the ER and mitochondria. This communication includes the transfer of lipids and sterols from the ER to mitochondria [25,26], as well as the supply of Ca^2+^ needed for the enzymatic activity of Krebs cycle dehydrogenases and, thus, mitochondrial energy production from oxidative phosphorylation (OXPHOS) [27].

In the case of ALS, the genetics of patients have identified a surprising array of functions controlled by the products of mutated genes. ALS, also known as Lou Gehrig’s disease, is a spectrum of particularly aggressive neurodegenerative diseases that typically result in death within 2–5 years of diagnosis from respiratory failure [28]. The first description of ALS occurred in 1869 by the French neurobiologist Jean-Martin Charcot [29]. The incidence of ALS is 2–3 per 100,000 individuals, but these numbers show remarkable geographic differences [30,31]. For both spontaneous and familial ALS (sALS, fALS), the average age onset is between 40 and 60 years of age [31]. Ninety percent of ALS patients suffer from sALS, which tends to have a later onset than fALS [32]. The symptoms of ALS include muscle weakness, spasticity, hyperreflexia, fasciculations, dysarthria, dysphagia, and eventually respiratory impairment associated with the selective death of motor neurons in the motor cortex, brainstem, and spinal cord [33,34]. In 1993, a breakthrough finding identified superoxide dismutase 1 (SOD1) as the product of the *ALS1* gene leading to fALS [35]. This suggested disrupted control of redox conditions is a factor in this disease [36], since SOD1 is a superoxide scavenger that localizes to multiple compartments, including the cytosol and mitochondria [37]. Numerous point mutations of *SOD1* are connected to ALS, but there is no clear loss of function pattern associated with these mutant proteins [38]. Rather, gain of function effects dominate for mutant proteins and result in the aggregation of SOD1 clusters within astrocytes or motor neurons [39,40]. In these cell types, SOD1 aggregates are found in the cytosol, but also within mitochondria, thus creating oxidative stress [41,42]. Other ALS gene products have been identified that produce proteins controlling mitochondrial protein import (coiled-coil-helix-coiled-coil domain protein 10, CHCHD10, [43]), the cytoskeleton (e.g., Profilin-1, *ALS18*), mRNA stability (e.g., Fused in sarcoma, FUS, *ALS6*), and protein trafficking (e.g., valosin-containing protein, VCP, *ALS14*) [44,45].

Mutations in these genes give rise to defective proteins that trigger the ALS pathology exclusively within motor neurons, consistent with the historic view of neurodegenerative diseases [46]. However, such a narrow cellular point of origin of ALS today appears unlikely. Genetic analyses published this year show that GABAergic interneurons and oligodendrocytes are also consistently showing transcriptomic changes in ALS [47]. Moreover, in addition to classic ALS, which affects both upper and lower motor neurons, subtypes can involve the degeneration of specific populations of motor neurons [48]. Together, a complex picture emerges where multiple genetic changes in a variety of cell types compromise the normal functioning of the CNS, and seemingly without a clear-cut cell biological link.

## 2. Important ALS Genes and Cytosolic Stress

Today, in addition to *SOD1*, more than 30 further genes are known whose mutations can give rise to ALS. Together, these genes account for about 15% of fALS [49,50] and we the ones relevant for this review in Table 1.

The most common genetic abnormalities associated with fALS are hexanucleotide repeat expansions in the intronic region of *C9orf72* [51,52], which together with the mutations in *SOD1*, account for around 30–50% of fALS cases [45]. The large expansions of a non-coding GGGGCC-repeat in the first intron of the *C9orf72* gene form highly stable RNA quadruplexes, which can cause toxicity by at least three different mechanisms [53]: (1) They can influence general RNA transcription, splicing, translation and transport [54,55]. (2) They can result as toxic in their own right through the production of dipeptide repeats (DPRs) [56,57], which are derived from repeat-associated non-AUG (RAN) translated mRNAs [58,59]. These DPRs interfere with nucleocytoplasmic transport [60]. (3) DPRs reduce the amounts of the *C9orf72* transcript and protein [61], which exacerbates DPR toxicity further [62]. This latter observation raises the question on the endogenous function of the *C9orf72* protein product. The C9orf72 protein localizes to the cytosol [63], the endo-lysosomal system [64], and mitochondria [65]. In these compartments, several roles for C9orf72 have been identified: One function involves the association of C9orf72 with Smith–Magenis syndrome chromosomal region candidate gene 8 (SMCR8) and WD40 repeat-containing protein 41 (WDR41). This protein complex initiates autophagy by recruiting the ULK1 kinase and inducing phagophore formation [64]. Subsequently, C9orf72 acts as a GDP/GTP exchange factor (GEF) with its partners to activate Rab proteins such as Rab8a and Rab39b [66] and initiate autophagy within dendrites through the ULK1 complex [67]. This neuron-specific role of autophagy helps maintain dendritic arborization [67]. Within mitochondria, C9orf72 acts as a chaperone for the first complex of the OXPHOS electron transport chain [65]. Consistent with these two distinct roles in the suppression of ROS and toxicity, the reduction of C9orf72 protein levels results in increased ROS and inflammation [68]. Together, these findings identify a shared role of SOD1 and C9orf72 as guardians of cellular redox homeostasis. Thus, oxidative stress and inflammation are a direct consequence of their mutation in ALS, resulting in protein aggregation and disrupted homeostasis of their own mRNA and cellular mRNAs overall.

Amongst the more than 30 ALS genes, additional important contributors to the number of ALS patients are found in the *FUS* gene-encoding form fused in sarcoma/translated in liposarcoma (FUS/TLS) [69], and in the *TARDBP* gene-encoding transactive response DNA binding protein 43 (TDP-43) [70,71]. These two genes contribute another 7% of fALS cases and also contribute to sALS [45]. Both proteins can bind DNA and when they do so, they act to prevent DNA damage [72]. Moreover, both proteins regulate RNA metabolism including mRNA splicing and transport [73]. The toxicity of TDP-43 and FUS/TLS proteins is linked to their altered intracellular localization. Thus, TDP-43 normally localizes to the nucleus, but in cells from 97% of ALS patients, TDP-43 is found in cytoplasmic aggregates in a hyperphosphorylated form [74], which is toxic for motor neurons [75]. Within these aggregates, TDP-43 is ubiquitinated and cleaved [71]. This indicates that this pathological change is a key hallmark of ALS. As a result of the reduced activity of TDP-43, the expression of hundreds of gene products that are critical for the functioning of motor neurons is reduced [76]. The protein most reduced is Golgi-associated Stathmin-2 [77], a microtubule-destabilizing protein that results in being truncated and dysfunctional upon TDP-43 aggregation. As a consequence, neurite outgrowth decreases [78,79]. 

In ALS patient CNS cells, FUS is similarly found in the cytoplasm [80]. Like in the case of TDP-43, FUS mislocalization to cytoplasmic inclusions leads to a loss of function within the nucleus [81], but toxic gain of function in the cytoplasm is another consequence. Here, FUS aggregates alter expression of a set of mRNAs distinct from the *TDP-43*-controlled transcripts [82]. Overall, despite their ubiquitous defects in ALS, the mutations of *TARDBP* and *FUS* appear to create similar effects that trigger the pathology under specific circumstances.

## 3. Aggregation-Derived Cytosolic Stress Triggers ER Dysfunction

An important consequence of TDP-43 and FUS aggregates is the decreased ability of the cytoplasm to buffer against oxidative stress. This effect derives from an altered mitochondrial proteome, which fuels a feed-forward loop upon initiation of oxidative stress [83]. To a significant extent, the consequences of these stress-related effects appear to converge on the ER. This convergence on the ER could be shared between ALS and AD. For instance, mutant TDP-43, FUS, and SOD1 proteins disrupt the reticulon-4-dependent transport of soluble amyloid precursor proteins (sAPP) along ER tubules in the neuromuscular junction, suggesting ER structure and function is disrupted in neurodegeneration [84]. As a consequence, such a structural disruption of the ER, for instance via the depletion of atlastins, triggers the UPR [85]. This leads to the increased transcription of chaperones and an inhibition of general mRNA translation. Moreover, misfolded proteins are retrotranslocated from the ER to the cytosol, where they undergo ER-associated degradation (ERAD) [86]. This degradation process is initiated by the binding of ER chaperones and lectins such as the ER degradation-enhancing α-mannosidase-like protein (EDEM) to misfolded proteins [87,88]. Next, ERAD adaptors including Gp78/autocrine motility factor receptor (AMFR) transfer substrates to a retrotranslocation channel [89,90] from specific domains of the ER [91]. Here, cytosolic valosin-containing protein (VCP)/p97 mediates their extraction from the ER membrane [92], followed by degradation via the proteasome [86], implicating defective ERAD in the ALS pathology. 

When such protective mechanisms like ERAD fail, Ire1 promotes cell death through the activation of c-jun N-terminal kinase (JNK) [93]. Similarly, PERK activates pro-apoptotic transcriptional responses mediated by its downstream targets activating transcription factor 4 (ATF4) and the pro-apoptotic transcription factor C/EBP homologous protein (CHOP) [94]. This leads to the induction of pro-apoptotic proteins of the Bcl-2 family, including Bim and Puma [95,96]. In parallel, ER redox conditions change during oxidative stress [97,98]. A key change is an increase of protein disulfide isomerase (PDI) protein levels in most neurodegenerative syndromes as part of the UPR [99]. Interestingly, however, this increased amount of PDI is associated with its inactivation via S-nitrosylation, which exacerbates the toxicity of protein aggregates [100]. This oxidative inactivation of PDI increases in the presence of the mutant *SOD1* gene product [101]. Consistent with this link, the replication of patient-derived mutations of the *PDI* gene product and of the closely-related ERp57 oxidoreductase cause motor neuron dysfunction in a zebrafish model [102], suggesting that altered redox buffering within the ER can form a mechanistic basis for ALS.

Such changes within the ER occur early during disease onset, as shown by mutant *SOD1* G93A transgenic mice that show ER stress as early as postnatal day 5, preceding the onset of more classic symptoms [11]. The observed activation of the Ire1 branch of the UPR appears to accelerate disease progression. This is shown by delayed onset of ALS symptoms in mutant *SOD1* mice with XBP1 deficiency in parallel with autophagic degradation of toxic SOD1 aggregates [103]. In contrast, sustained PERK signaling as observed in *SOD1*-G85R mice prevents mutant SOD1 aggregation [104]. Accordingly, extending the PERK protective response pharmacologically with guanabenz, an alpha-2 adrenergic receptor agonist [105], measurably delayed ALS progression in a small-scale clinical trial [106]. In contrast, PERK haploinsufficiency enhances mutant SOD1 aggregation but does not decrease the induction of ATF4 at the early symptomatic stage [107]. This provides evidence that targeting the PERK pathway is not a promising strategy against the ALS pathology. Moreover, these results suggest a dichotomic role for ER stress, based on Ire1 and PERK. The UPR activation of Ire1 therefore appears to accelerate ALS in early stages, but PERK acts as a protective factor. Potentially, these divergent findings could derive from distinct vulnerability of cell types to this ER signaling pathway that could preclude general applicability [11]. Alternatively, the role of ER stress as a controller of cell metabolism could explain some of these findings. This attractive hypothesis is based on the role of ER stress in the modulation of ER-mitochondria Ca^2+^ flux [108], which activates mitochondria ATP production upon activation of the UPR [109].

## 4. ALS-Associated Stress Signaling Converges on Membrane Contact Sites

**Figure 1:**
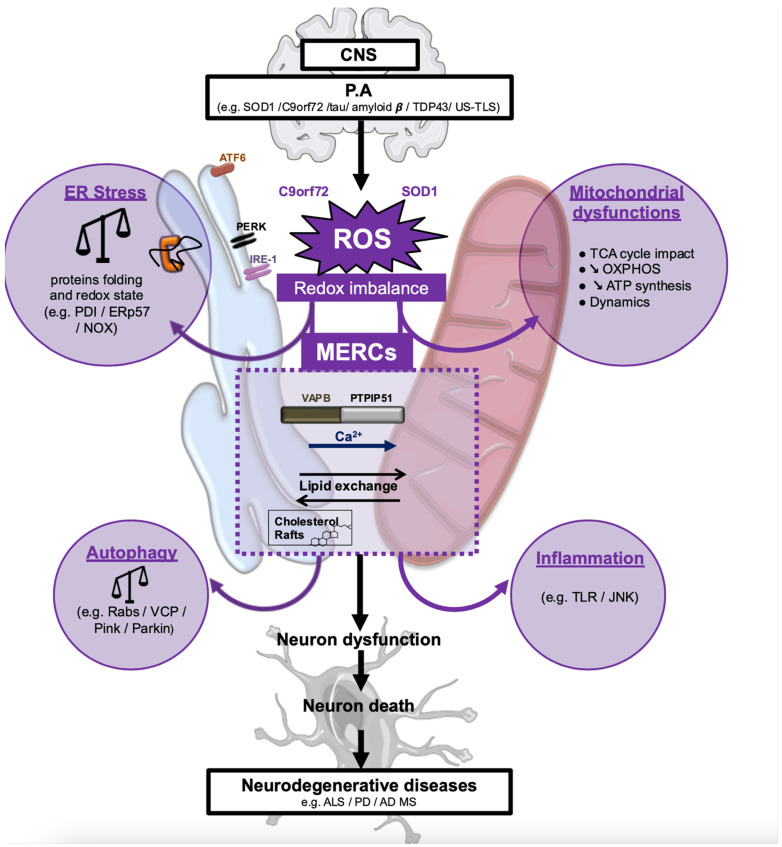
Overview on the convergence of pathological mechanisms on the mitochondria-ER contacts (MERCs). Protein aggregates (P.A.) cause reactive oxygen stress, which modulates MERCs and disrupts their physiological control of mitochondrial bioenergetics, ER stress, autophagy, and inflammation, resulting in the neurodegenerative pathology.

The majority of studies on the effects of ALS-connected mutations address local effects of the resulting mutant proteins. Typical examples are the consequences of mutant SOD1 protein for the functioning of mitochondria and of mutant TDP-43 protein for nuclear gene expression. However, cellular organelles are working in a continuum and other neurodegenerative syndromes, in particular AD, show defective functions of contacts between organelles [110], most prominently between the ER and mitochondria [111,112,113,114,115] (Figure 1). This important observation raises the question whether ALS progression also depends on changes of inter-organellar interactions. Such a role for MCS in ALS is supported by observed changes of the ER and Golgi structure accompanied by mitochondrial crystalline particles found within patient tissue, suggesting the functional relationships between these organelles change [116,117]. Over the past decade, MCS have received a lot of attention as determinants of organellar function and homeostasis [15]. An important member of MCS are MERCs, which mediate a flux of Ca^2+^ and lipids between the two organelles that control mitochondrial functions including OXPHOS and apoptosis [118]. The function of MERCs was recently reviewed extensively by us and other labs and the reader is referred to those texts [119,120,121].

In the ALS context, oxidative stress, as derived from mutant SOD1 that leads to high concentrations of reactive oxygen species (ROS), influences the metabolic functions of MERCs as promoters of mitochondrial oxidative phosphorylation (OXPHOS) [122]. Thus, ROS from ALS-associated protein aggregation can trigger or exacerbate mitochondrial oxidative stress [123]. Mechanistically, this oxidative stress is a consequence of altered MERC structures that trigger physiological changes due to shortages of key metabolites needed for OXPHOS [124]. Detrimental to the functioning of CNS cells, as a consequence, these mitochondrial ROS further increase protein aggregation [125]. Therefore, a vicious cycle is set in motion, as protein aggregates can interfere with mitochondrial function [126] or enzymes of the NADPH oxidase family to further increase ROS content within affected cells [127]. Other ways how protein aggregates induce the production of ROS are the exhaustion of cellular chaperones such as Hsp70 [128], damage to membranes including the formation of pores [129], and altered functions and interactions of such membranes, notably of MERCs formed between the ER and mitochondria [130].

Consistent with a role of MERCs in ALS, mutations in the gene-encoding vesicle-associated membrane protein (VAMP)-associated protein B (VAPB) have been found in cases of fALS [131,132]. VAPB was first described as an ER-associated protein that controls coatomer-mediated ER-Golgi trafficking [133]. Later, it emerged as an important ER membrane tethering protein via interaction with plasma membrane Kv2 potassium channels [134,135] and with mitochondrial tyrosine phosphatase interacting protein of 51 kDa (PTPIP51) [136]. Typically, VAPB acts as a MCS-forming tether through recognition of a motif consisting of two phenylalanines (FF) in an acidic tract (FFAT) in its partners [137]. This motif allows the major sperm protein (MSP) domain of VAPB to nucleate a scaffold between the ER and any proximal organelle where a FFAT-containing protein is found [138]. Potentially, the interactions between VAPB and partner proteins can depend on the phosphorylation of these FFAT motifs [139].

As a tether, VAPB controls multiple functions in CNS cells including protein transport, lipid metabolism, and the UPR [140]. Like many ALS gene products, this equips VAPB with far-ranging influence, extending to key mechanisms in neurodegeneration like autophagy [22], or the uptake of Ca^2+^ by mitochondria [136]. Interestingly, disrupted mitochondrial and MERC functions from mutant *VAPB* could also be a factor in other types of neurodegeneration like AD [141]. The consequence of mutant *VAPB* is a reduction of mitochondrial OXPHOS that normally depends on Ca^2+^ import from the ER for its dehydrogenases and the ATPase [118]. While VAPB also interacts with peroxisomal ACBD5 to mediate tethering of the ER to peroxisomes [142,143], the connection of this function to dysregulation of peroxisomal lipid metabolism [144] or ALS is currently not known. Some of the effects of mutant VAPB may derive from its role as a tail-anchored adaptor for MERC-localized lipid transfer [145]. Thus, mutant VAPB alters ER lipid composition, and creates an electron-dense restructured ER [146], characterized by inclusions and organized smooth ER [131]. However, its role in tethering may not be limited to the formation of MERCs that require lipid rafts, but could radiate out to additional compartments downstream in the secretory pathway. Interestingly, the tethering function of VAPB between the ER and mitochondria is disrupted upon toxic gain of function of mutant FUS at MERCs [147].

Another function of VAPB is a type of autophagy that is also referred to as mitophagy. This degradative mechanism eliminates dysfunctional mitochondria and increases in the presence of mutant VAPB, but only if this pathway is triggered by rapamycin and not general starvation [22]. Thus, increased autophagy could either cause the elimination of functional mitochondria due to mutant *VAPB* or due to the accumulation of dysfunctional mitochondria exhibiting low bioenergetics [148]. This latter scenario can also be triggered by a mutation in a gene encoding optineurin, which is also implicated in ALS [149,150] and which acts as an autophagy receptor for damaged mitochondria [151].

Quite a few additional ALS gene products are MCS regulatory proteins. For instance, the *ALS2* gene Alsin [152] suppresses SOD1 toxicity [153]. Alsin is a GEF for the small GTPase Rab5. Under conditions of mitochondrial oxidative stress, endosomal Rab5 moves onto endosome-mitochondria contacts, where it forms a complex with Alsin to direct lipids from endosomes to mitochondria, thus maintaining mitochondrial membrane properties [154]. In fruit flies, mutant Alsin also impacts the functions of endosomes in the subsynaptic reticulum, thus compromising synaptic development and neuronal survival [155].

Another example is *VCP*, which is responsible for 1–2% of fALS cases [156]. The *VCP* gene product acts as a ubiquitin-specific chaperone, controlling proteasomal protein degradation at the ER [157], mitochondria [158] and lysosomes [159]. On ER-mitochondria contacts, it interacts with the E3 ubiquitin ligase Gp78/AMFR, which in addition to its role in ERAD also determines MERC tethering through two functions: on the one hand, it controls mitofusin-2 amounts [160,161]. On the other hand, Gp78/AMFR also mediates degradation of HMG-CoA reductase, one of the enzymes of the mevalonate/cholesterol pathway [89], and thus controls cholesterol levels at the ER that are required for MERC cholesterol raft formation [162]. In the presence of mutant VCP protein, MERCs are thus expected to be dysfunctional, due to altered cholesterol levels. Moreover, the mutation of *VCP* is expected to disrupt the control of OXPHOS normally maintained by Ca^2+^ and ROS signaling on MERCs, an observation that is indeed made in patient fibroblasts [163]. Given normal MERCs are an important point of origin for autophagosomal membranes [164], mutant *VCP* also leads to reduced autophagic clearance of TDP-43 and FUS aggregates [165,166], potentially through Beclin-1-dependent autophagy initiation [167] or through the inhibition of the PINK/Parkin pathway [168]. Similar to the findings with VAPB, *VCP* mutations are associated with disrupted metabolic signaling between the ER and mitochondria [169].

MERC dysfunction in ALS can also occur more indirectly. For instance, the candidate ALS gene product CHCHD10 is a mitochondrial intermembrane space (IMS) protein with two Cys-X9-Cys motifs. CHCHD10 controls redox in the IMS together with the oxidoreductase CHCHD4 (known in yeast as Mia40) and the sulfhydryl oxidase ALR (known in yeast as Erv1) to catalyze the correct formation of disulfide bonds of IMS and inner mitochondrial membrane (IMM) proteins [170]. Through this central role for the homeostasis of mitochondrial membrane proteins, CHCHD10 maintains OXPHOS, which is critical for energy homeostasis within the ER, thus preventing UPR induction in both organelles [171].

This pattern is confirmed with yet another ALS gene, the Sigma-1 receptor (SIGMAR1) [172]. This MERC-associated chaperone maintains the Ca^2+^ signaling machinery in the form of IP_3_Rs at the contact site [173]. *ALS16*-mutant SIGMAR1 is incapable of stabilizing IP_3_Rs at MERCs, which causes the breakdown of metabolic signaling towards mitochondria and a reduction of mitochondrial ATP production. Interestingly, this mechanism is prominently associated with motor neurons in the CNS, thus potentially providing a mechanistic basis for the specificity of the affected cell types [174]. An additional consequence is defective autophagy and ER stress [175], two known consequences of MERC disruption [119].

This latter observation returns our focus on ER stress. The mechanistic links between this condition and MCS include the sensor proteins Ire1 and PERK. Interestingly, unlike their functions in ER stress that have opposing consequences in ALS, both act to maintain MERC signaling [176] and formation [177]. This could suggest these transmembrane stress sensors are attractive candidates for yet-to-be-discovered mutations in ALS.

## 5. MERCs in Other Types of Neurodegeneration

The disruption of normal mitochondrial functions and the aggregation of proteins are both a target and a source of CNS oxidative stress that also contributes to disease progression in other types of neurodegeneration. It appears plausible to weigh their relative contribution to the disease etiology via the assessment of genetic factors involved. Thus, some types of neurodegeneration clearly have their dominant origin in the toxic accumulation of protein aggregates, such as AD. The genetics of these diseases show mutations in genes giving rise to aggregates formed by cytosolic proteins like APP [178]. However, in parallel or even preceding such AD-associated protein aggregation, mitochondrial dysfunction increases measurably, seen for instance as glucose hypometabolism on positron-emission tomography of the CNS [179,180]. These changes are accompanied by alterations in mitochondrial metabolic enzymes, such as increased activity of Krebs cycle dehydrogenases like succinate dehydrogenase that produces fumarate [181]. Another early hallmark of AD is the decrease of OXPHOS enzymes [182], which are typically under control of redox-dependent chaperones localized to the IMS [183]. This suggests a loss of the metabolic equilibrium within these mitochondria, which is potentially tied to oxidative stress.

An interesting consequence of the classification of neurodegenerative syndromes as general protein misfolding disorders [184] is the activation of ER stress signaling from the formation of protein aggregates in the cytoplasm of CNS cells [6]. While neurofibrillary tangles composed of β-amyloid and phosphorylated tau accumulate in the cytosol of affected cells of the CNS [185,186], they are hypothesized to also trigger the ER-specific UPR [187]. Mechanistically, this ER stress signaling could be initiated due to the accumulation of β-amyloid, following its generation by the γ-secretase protease complex within MERCs [188]. As discussed above in the case of ALS, the presence of these bulky aggregates appears to disrupt the normal interaction of the ER with mitochondria, followed by the initiation of the UPR signaling mechanism also in AD [189,190]. Another mechanistic explanation for this observation is based on an activity of presenilins (PS) as Ca^2+^ modulatory proteins [190,191]. These two ER transmembrane proteins (PS1 and PS2) are AD genes and are best known for their role as components of γ-secretase [192]. Mutant PS1 and PS2 increase the 42-residue form of β-amyloid [193]. However, both PS1 and PS2 are also able to reduce ER Ca^2+^ content [189,190], likely through a modulation of IP_3_R Ca^2+^ release [194]. This could increase the tethering between the ER and mitochondria, due to the decreased speed of moving mitochondria in the presence of high [Ca^2+^] [195]. The resulting functional link has led to the proposal that aberrant MERC formation initiates AD [110]. According to this model, AD is a consequence of dysfunctional MERCs, including increased Ca^2+^ flux towards mitochondria, increased cholesterol raft formation, oxidative stress, and disrupted bioenergetics, a hypothesis first formulated in 2010 by Eric Schon and Estela Area-Gomez [111]. Consistent with this idea, lipid raft formation on these contact sites dramatically increases upon accumulation of the 99-amino acid C-terminal fragment of APP [115]. Elegant studies have detected altered mitochondrial bioenergetics in a number of familial AD cell models [196], which is a key consequence of altered ER–mitochondria interaction [118].

Like in ALS, the ERAD pathway results as dysfunctional in many types of neurodegeneration [197]. As a consequence, events taking place in the cytosol can further increase ER stress. Moreover, it is likely that this ER stress is a factor in the development of neuroinflammation due to its control over the production of inflammatory cytokines [198,199]. Importantly, it is also a hallmark of neurodegeneration associated with neuroinflammation [200], where ER stress also cross-talks with mitochondrial membrane dynamics [201]. Together, the formation of cytosolic protein aggregates in neurodegenerative syndromes can mechanistically trigger mitochondrial dysfunction, altered ER–mitochondria interaction (a key characteristic of AD), ER stress, and the inflammation of the CNS. These triggers are interconnected in a web-like manner and subsequently trigger apoptotic and necrotic mechanisms, thus leading to the neurodegenerative pathology.

On the other end of the spectrum is PD, where genes encoding mitochondrial proteins feature more prominently, albeit alongside other genetic factors [202]. In the case of PD, almost 90 genes are known to cause the monogenic disease type [203]. While dopaminergic neurons are thought to be the first target of the disease [204], single-cell transcriptomic data has recently identified changes in enteric neurons and oligodendrocytes as an additional hallmark of PD [205]. Several PD gene products determine mitochondrial functions and quality control by controlling and executing the autophagic elimination of these organelles [206]. Within dopaminergic neurons, these mitochondrial dysfunctions occur in parallel with cytosolic protein aggregates of α-synuclein, also called Lewy bodies, especially if these latter are found close to mitochondria [207] or within the mitochondrial membrane of dopaminergic neurons [208]. Some aggregates also localize to MERCs, structures that are necessary to maintain mitochondria membrane dynamics and metabolism [209]. Oxidative stress from these aggregates is detected by DJ-1/PARK7, which undergoes cysteine oxidation and promotes antioxidant stress gene production via the transcription factor Nrf2 [210,211]. Central to PD is the disruption of autophagic elimination of dysfunctional mitochondria. Normally, this mechanism is controlled by PTEN-induced kinase 1 (PINK1/PARK6) and Parkin (PARK2), but several additional gene products associated with mitochondria directly control disease progression and are known as *bona fide PARK* genes [212]. Together with PINK1, these gene products control, for instance, mitochondrial dynamics (LRRK2/*PARK8*, VPS35/*PARK17*) [213,214]. Another example is the coiled-coil-helix-coiled-coil-helix domain containing protein 2 (CHCHD2/*PARK22*), which is a nuclear-encoded mitochondrial protein that partially localizes to the IMS [215], from where it controls mitochondria movement along microtubules [216], the anti-apoptotic activity of mitochondrial Bcl2 family proteins, as well as the cleavage of Opa1, a GTPase that normally mediates the fusion of IMM [217,218]. These activities likely require cooperation with CHCHD10 [219]. Interestingly, mutations in these IMS proteins are found mutated in both ALS and PD [220]. Also relevant for our review article, PD-mutant proteins control mitochondrial Ca^2+^ import from the ER needed for mitochondrial metabolism (e.g., DJ-1/*PARK7*) [221]. The ER-stress-induced GTPase Rab32 [201] (p.Ser71Arg mutation) that controls localization of Ca^2+^ handling proteins and dynamin-related protein 1 (Drp1) to MERCs [222] is also amongst this group [223], potentially as an upstream regulator of LRRK2 [224]. The central role of mitochondria in the PD pathology receives further support from the role of OXPHOS inhibitors rotenone and oligomycin, which trigger reversible α-synuclein aggregation and lead to PD-like symptoms in animal models [225,226].

## 6. Conclusions

**Figure 2:**
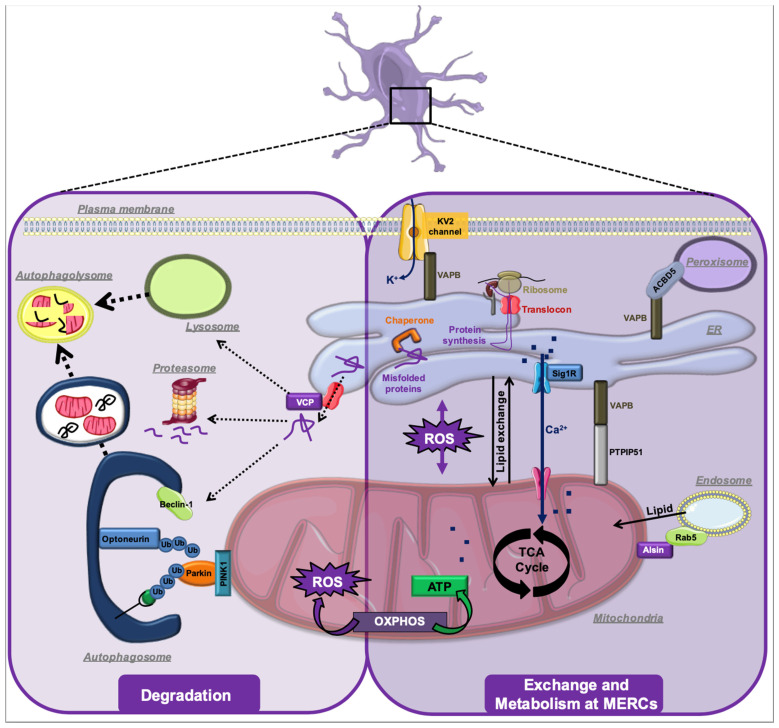
The context of MERC-associated ALS-related MCS proteins. For details, see text.

A change of the intracellular architecture, where various organelles no longer interact normally, appears to constitute a novel hallmark of several neurodegenerative diseases, notably of ALS (Figure 2). These diseases, therefore, emerge as “membrane contact site (MCS) pathologies”. The formation and consequences of various protein aggregates remain important factors, and like in the examples of AD and PD, future research will have to address the causative relationship between these aggregates and MCS dysfunction. Further research will also have to determine which one(s) and how many of the MCS control the progression of neurodegeneration. In the case of ALS, MERCs appear critical (Figure 2), given many MERC-controlling proteins are amongst the ALS gene products. However, alternatively, a general imbalance of MCS could be a key factor also in this pathology.

## Figures and Tables

**Table 1:** MCS-associated genes mutated in ALS.

Locus	Gene	Protein	Protein Function	Role in ALS
21q22.1	*SOD1*	Superoxide dismutase-1 (SOD1)	UPR activation ERAD inhibition Antioxidant defense Autophagy enhancement	Mutants cause toxic SOD1 clusters aggregation within astrocytes or motor neurons.
2q33.2	*ALS2*	Alsin	A guanine-nucleotide exchange factor (GEF) to activate the GTPase Rab5.	Mutants influence the functions of endosomes in the subsynaptic reticulum.
9p13-p12	*VCP*	Valosin-containing protein (VCP) or Transitional endoplasmic reticulum ATPase (TER ATPase)	Ubiquitin/protein degradation Secretory protein trafficking	Mutants disrupt the control of OXPHOS and reduce autophagic clearance of TDP-43 and FUS aggregates.
10p13	*OPTN*	Optineurin	Selective autophagic adaptor Protein aggregates clearance	Mutants (e.g., E478G, R96L) are associated with both fALS and sALS.
20q13.3	*VAPB*	Vesicle-associated membrane protein (VAMP)-associated protein B (VAPB)	Vesicle trafficking ATF6 sensor interaction and XBP1 inhibition Lipid metabolism Microtubule organization	A dominantly inherited mutant, P56S-VAPB, causes fALS.
9p13.3	*SIGMAR1*	Sigma-1 receptor (SIGMAR1)	Calcium signaling Lipid metabolism	A missense mutation in *SIGMAR1* (e.g., G304C) causes fALS; Lack of *SIGMAR1* induces motoneuron hyperexcitability and exacerabates ALS pathology.
16p11.2	*FUS*	Fused in sarcoma (FUS)	Transcriptional activation Protein and RNA binding	Mutations in *FUS* cause fALS and lead to the cytosolic deposition of FUS in the brain and spinal cord of ALS-FUS patients.
1p36.2	*TARDBP*	TAR-DNA binding protein (TDP43)	Transcriptional repression DNA and RNA binding	*TARDBP* gene rearrangement has been implicated in the pathogenesis of ALS; Mutations in the *TARDBP* gene (e.g., M337V and Q331K) are related to ALS.
22q11.23	*CHCHD10*	Coiled-coil-helix-coiled-coil domain protein 10 (CHCHD10)	Mitochondrial Cristae morphology maintenance Oxidative phosphorylation	Mutants are associated with ALS as well as in other mitochondrial diseases.
17q25	*P4HB*	Protein disulfide-isomerase A1 (PDIA1)	Disulfide bonds formation, breakage and rearrangement Inhibition of misfolded proteins aggregation	Together with other ER stress markers, PDIA1 is greatly elevated in ALS spinal cord.

## Data Availability

Not applicable.
